# Multimodality Imaging in Advanced Heart Failure for Diagnosis, Management and Follow-Up: A Comprehensive Review

**DOI:** 10.3390/jcm12247641

**Published:** 2023-12-12

**Authors:** Valeria Pergola, Matteo Cameli, Giulia Mattesi, Saima Mushtaq, Antonello D’Andrea, Andrea Igoren Guaricci, Maria Concetta Pastore, Filippo Amato, Carlo Maria Dellino, Raffaella Motta, Martina Perazzolo Marra, Santo Dellegrottaglie, Roberto Pedrinelli, Sabino Iliceto, Savina Nodari, Pasquale Perrone Filardi, Gianluca Pontone, on behalf of the Cluster Imaging of Italian Society of Cardiology (SIC)

**Affiliations:** 1 Department of Cardiac, Thoracic and Vascular Sciences and Public Health, University of Padua, Via Giustiniani 2, 35128 Padova, Italy; giulia.mattesi@aopd.veneto.it (G.M.); filippo.amato@studenti.unipd.it (F.A.); martina.perazzolomarra@unipd.it (M.P.M.); sabino.iliceto@unipd.it (S.I.); 2 Department of Cardiovascular Diseases, University of Sienna, 53100 Siena, Italy; matteo.cameli@unisi.it (M.C.); mariaconce.pastore@unisi.it (M.C.P.); 3 Department of Perioperative Cardiology and Cardiovascular Imaging, Centro Cardiologico Monzino IRCCS, 20138 Milan, Italy; saima.mushtaq@cardiologicomonzino.it (S.M.); carlo.dellino@humanitas.it (C.M.D.); gianluca.pontone@unimi.it (G.P.); 4 Cardiology Unit, Umberto I Hospital, 84014 Nocera Inferiore, Italy; antonellodandrea@libero.it; 5 University Cardiology Unit, Interdisciplinary Department of Medicine, Policlinic University Hospital, 70121 Bari, Italy; andreaigoren.guaricci@uniba.it; 6 Unit of Radiology, Department of Medicine, Medical School, University of Padua, 35122 Padua, Italy; raffaella.motta@unipd.it; 7 Division of Cardiology, Ospedale Medico-Chirurgico Accreditato Villa dei Fiori, 80011 Acerra, Italy; sandel74@hotmail.com; 8 Cardiac, Thoracic and Vascular Department, University of Pisa, 56126 Pisa, Italy; roberto.pedrinelli@unipi.it; 9 Department of Medical and Surgical Specialties, Radiological Sciences, and Public Health, Institute of Cardiology, University of Brescia, 25123 Brescia, Italy; savina.nodari@unibs.it; 10 Department of Advanced Biomedical Sciences, Federico II University of Naples, 80138 Naples, Italy; pasquale.perrone@unina.it; 11 Department of Biomedical, Surgical and Sciences, University of Milan, 20122 Milan, Italy

**Keywords:** multimodality imaging, advanced heart failure, extracorporeal cardiac support, cardiac transplant

## Abstract

Advanced heart failure (AHF) presents a complex landscape with challenges spanning diagnosis, management, and patient outcomes. In response, the integration of multimodality imaging techniques has emerged as a pivotal approach. This comprehensive review delves into the profound significance of these imaging strategies within AHF scenarios. Multimodality imaging, encompassing echocardiography, cardiac magnetic resonance imaging (CMR), nuclear imaging and cardiac computed tomography (CCT), stands as a cornerstone in the care of patients with both short- and long-term mechanical support devices. These techniques facilitate precise device selection, placement, and vigilant monitoring, ensuring patient safety and optimal device functionality. In the context of orthotopic cardiac transplant (OTC), the role of multimodality imaging remains indispensable. Echocardiography offers invaluable insights into allograft function and potential complications. Advanced methods, like speckle tracking echocardiography (STE), empower the detection of acute cell rejection. Nuclear imaging, CMR and CCT further enhance diagnostic precision, especially concerning allograft rejection and cardiac allograft vasculopathy. This comprehensive imaging approach goes beyond diagnosis, shaping treatment strategies and risk assessment. By harmonizing diverse imaging modalities, clinicians gain a panoramic understanding of each patient’s unique condition, facilitating well-informed decisions. The aim is to highlight the novelty and unique aspects of recently published papers in the field. Thus, this review underscores the irreplaceable role of multimodality imaging in elevating patient outcomes, refining treatment precision, and propelling advancements in the evolving landscape of advanced heart failure management.

## 1. Introduction

According to the European Society of Cardiology (ESC) and the American College of Cardiology (ACC)/American Heart Association (AHA)/Heart Failure Society of America (HFSA) Guidelines, advanced heart failure (AHF) is recognized as a clinical syndrome characterized by signs and symptoms of volume overload and inadequate blood perfusion despite maximal therapy, resulting in recurrent hospitalizations and high mortality [1,2]. AHF is further defined by marked symptoms significantly impacting daily life, leading to recurrent hospitalizations despite attempts to optimize guideline-directed medical therapy [2]. The prevalence of AHF is on the rise due to an aging population, improved treatment, and increased survival from HF [3].

Despite medical advancements, patients with AHF still face a poor prognosis, with 1-year mortality rates ranging from 25% to 75% [1]. Additionally, they suffer recurrent episodes of pulmonary or systemic congestion, low cardiac output, and malignant arrhythmias, leading to at least one unplanned hospitalization per year [4].

AHF patients typically exhibit severe exercise intolerance, as evidenced by parameters such as a 6-min walking test distance of <300 m or peak oxygen consumption (pVO2) <12 mL/kg/min or <50% of the predicted value [5]. Furthermore, recent evidence highlights that a significant proportion of patients with AHF exhibit mildly reduced or preserved left-ventricular ejection fraction (LVmrEF and LVpEF, respectively), and their survival outcomes are poor irrespective of EF [6,7]. The challenge in comprehending AHF lies in the absence of a singular diagnostic criterion, thereby complicating the establishment of a universally applicable case definition within diverse populations.

Patients at this stage of HF often exhibit poor responses to conventional therapies, including optimal medical management, cardiac resynchronization therapy (CRT), and various percutaneous and surgical interventions aimed at addressing valvular and coronary artery issues. The clinical spectrum of AHF ranges from progressive refractory deterioration to cardiogenic shock [1]. While inotropic agents can provide temporary relief, their use is limited due to the risk of myocardial ischemia and tachyarrhythmias [8]. As per the latest guidelines from the European Society of Cardiology (ESC) for heart failure [1], treatment options may include adding sacubitril-valsartan or sodium-glucose co-transporter-2 (SGLT2) inhibitors, doubling the dose of loop diuretics, or combining them with thiazide-type diuretics such as metolazone. In cases of refractory diuretic treatment, renal replacement therapy should be considered [1].

The Interagency Registry for Mechanically Assisted Circulatory Support (INTERMACS) classification system plays a crucial role in assessing the severity of HF, ranging from class III NYHA (New York Heart Association) to critical cardiogenic shock, despite escalating therapeutic support [9]. For patients classified as INTERMACS 1 or 2, short-term mechanical circulatory support (MCS) devices are the preferred choice [10]. Therefore, when patients fail to stabilize with medical therapies alone, they become eligible for MCS devices, whether in acute or chronic settings. A variety of short- and long-term MCS devices are available to clinicians [10,11].

MCS devices can serve as a bridge to decision (BTD), bridge to recovery (BTR), bridge to other bridge therapies (BTB), such as long-term MCS, or as a bridge to urgent cardiac transplant (BTT) [11]. These devices encompass intra-aortic balloon pumps (IABP), mpella, veno-arterial extracorporeal membrane oxygenation (ECMO), and left-ventricular assist devices (LVADs). Regular imaging assessments, particularly echocardiography, are essential for monitoring device function, detecting complications, and optimizing patient outcomes. Imaging helps assess ventricular function, valve function, and the presence of any thrombus or device-related issues, ensuring timely intervention when necessary [1].

LVADs can be used as a BTT, a bridge to candidacy (BTC), or as permanent treatment, such as “destination therapy” (DT) (refractory HF, no transplant candidate), in order to overcome the shortage of heart donors [12]. In this setting, RV assessment is crucial since the RV supports the cardiac output and RV failure occurs in up to 50% of cases following LVAD implantation, resulting in high perioperative mortality and morbidity rates [13,14]. A biventricular assist device (BiVAD) is an implantable pump designed to help the heart function better when both the right and left pumping chambers of the heart are failing. However, BiVAD recipients have greater mortality and morbidity than LVAD recipients [15]. Therefore, MCS with BiVAD and total artificial heart (TAH) options remain challenging.

Orthotopic cardiac transplant (OCT) stands as the gold standard of care for eligible patients with advanced, refractory HF. OCT has demonstrated the ability to improve both the quality of life and overall survival [16]. However, the limited availability of suitable donor organs and the presence of numerous contraindications restrict the applicability of this option to a select group of patients [1]. Cardiac imaging is vital in the evaluation of transplant candidates. Echocardiography is the first-line imaging modality to assess the suitability of the donor’s heart. It also helps detect any potential contraindications such as valvular abnormalities, or ventricular dysfunction, which may influence the decision to proceed with transplantation [17].

Cardiac imaging modalities play a pivotal role in the assessment and management of AHF patients. They provide crucial insights into myocardial function and assist in identifying potential candidates for advanced therapies, selecting the most appropriate mechanical cardiac support (MCS) device, and optimizing its settings for individual patients [1,18].

Evaluating AHF through echocardiography has seen considerable growth, with an emphasis on hemodynamic and strain assessments. Nuclear techniques have also evolved, with innovations like positron emission tomography/computed tomography (PET/CT) and single-photon emission-computed tomography positron-emission tomography (SPECT), enhancing both diagnostic precision and the ability to assess myocardial blood flow (MBF) and viability. Meanwhile, cardiac computed tomography (CCT) imaging, already established for coronary disease evaluation, is increasingly valuable for characterizing myopathic conditions. Furthermore, cardiac magnetic resonance (CMR) is expanding its role in tissue characterization, now encompassing a broader range of diseases [18].

The purpose of this review is to provide a multimodality imaging approach for patients with AHF. Clinical decisions about HF management are frequently based on measurements of LV function, relying mainly on transthoracic and transesophageal echocardiographic (TTE and TEE) measurements. These tools are almost always available in primary care; this means that AHF clinical diagnosis and decision-making can take weeks, even months, of in-hospital stay and costly frequent visits to HF outpatient clinics. As a result, the opportunity for early detection of AHF is often lost. Currently, the availability of other imaging modalities, such as CCT, nuclear imaging, and CMR, in tertiary centers is of utmost importance in the assessment of complex scenarios, and this may have an impact on the survival of AHF patients. The aim of this review is to emphasize the novelty and unique aspects of recently published papers in the field, distinguishing it from previous reviews on this topic.

## 2. Multimodal Approach to Advanced Heart Failure

### 2.1. Transthoracic Echocardiography in AHF

The echocardiographic parameters used to evaluate in patients with AHF are:

1. Left-ventricular ejection fraction (LVEF). LVEF is a crucial indicator in assessing heart failure. In individuals with AHF, approximately 50% of patients may exhibit a reduced LVEF [1]. It is noteworthy, however, that half of AHF cases present with mildly reduced or preserved EF, with poor survival rates irrespective of EF values [6,7].
  The accurate measurement of LVEF is essential and can be achieved through the Simpson’ biplane method or 3D imaging. In instances of poor acoustic window quality, the use of ultrasound-enhancing agents (UEAs) is recommended to enhance the visualization of the endocardial borders [19].
  According to the 2022 AHA/ACC/HFSA Guideline, the diagnosis of HF with an LVEF exceeding 40% necessitates a demonstration of increased filling pressures. While increased cardiac filling pressure is presumed for HFrEF, individuals with HFmrEF or HFpEF require evidence demonstrating spontaneously or provokable increased LV filling pressures for a confirmed diagnosis of HF. Such supporting evidence can be obtained through noninvasive methods such as natriuretic peptide assessment or imaging for diastolic function [2]. Patients who initially had HFrEF and subsequently, at follow-up, show an LVEF surpassing 40%, are classified as having heart failure with improved EF (HFimpEF) [2].
2. Presence of regional wall motion abnormalities (RWMA). In AHF, the presence of RWMA serves as a significant clinical indicator, which may be caused by factors such as ischemia, scar tissue formation, or underlying structural heart disease. These abnormalities contribute to the overall dysfunction of the heart and can further compromise its pumping capacity [1,2].
3. Ventricular diameters and volumes: LV end-diastolic diameter (EDD) upper cut-off normal values are >52.2 mm in females and >58.4 mm in males. LV end-diastolic volume (EDV) upper cut-off values are >61 mm/m^2^ in the female sex and >74 mm/m^2^ in the male sex. Three-dimensional (3D) echocardiography is currently the most accurate technique in determining LV volume and function. It correlates with cardiac magnetic resonance, reducing the need for geometric assumptions. However, it has some limitations, such as lower spatial and temporal resolution [20]. In a retrospective study on 443 patients initially diagnosed with HFrEF, those with persistent EF ≤ 40% (HFprEF) at the 1-year follow-up had a poorer prognosis compared to those with HFimpEF. Notably, LV end-systolic diameter (LVESD) at discharge emerged as a significant predictor, with an LVESD ≥ 55 mm associated with a higher incidence of persistent HFrEF, suggesting its potential value for risk stratification in patients with advanced HF refractory for guideline-directed medical therapy [21]
4. Stroke volume (SV): the SV through the aortic valve is calculated as the product of the cross-sectional area times the integral of the velocity/time curve of flow through that area. The lower cut-off for indexed SV value is <35 mL/m^2^ [22]. Monitoring changes in indexed SV over time can provide insights into the progression or improvement of heart failure, guiding adjustments to therapeutic interventions.
5. LV global longitudinal strain (GLS). The normal value is strictly variable depending on sex and age, with a mean normal value of −22.5 ± 2.7 and a confidence interval = −17.2 to −27.7 [23]. In patients with HF with reduced LVEF, GLS is an accurate noninvasive measure of myocardial fibrosis and a better predictor of all causes of mortality than other echocardiographic parameters, especially in males and in sinus rhythm [24]. A GLS <16% has been proposed as a lower value for LV systolic dysfunction [2].
6. Mitral and tricuspidal regurgitation. TTE using quantitative parameters allows for the quantification of the seriousness of these valvular heart diseases, also being able to provide indications on the need for a possible percutaneous treatment. There are qualitative, semiquantitative, and quantitative parameters (Pisa radium, regurgitant volume (RV) and effective regurgitant orifice area (EROA)). For mitral regurgitation, the presence of EROA > 40 mm^2^ and Rvol > 60 mL is indicative of severity, while the severity cut-off for tricuspid regurgitation is: EROA > 40 mm, Rvol > 45 mL and Pisa radium > 9 mm [25];
7. Diastolic function. The E/E′ ratio > 14 and average e′ velocity < 9 cm/s identify an increase in LV filling pressure. During diastole, blood flows through the mitral valve when the LV relaxes, causing an early diastolic mitral velocity (E), and then additional blood is pumped through the valve when the left atrium contracts during late diastole (A). The E/A ratio can be altered as diastolic dysfunction progresses (with an initial decline (E/A < 1); then, there is a pseudonormalization (E/A ≥ 1) and, finally, a restrictive filling pattern (E/A ≥ 2) appears [26]. Tissue Doppler imaging is an echocardiographic technique that measures the velocity of the mitral annulus. This velocity has been shown to be an important marker of early myocardial dysfunction. With abnormal active relaxation, mitral annulus velocity during early diastole (e′) is decreased while mitral annulus velocity during late diastole (a′) is increased, resulting in a lowered e′/a′ ratio [27] and a higher E/e′ ratio. Indicators of increased filling pressures include an average E/e′ ≥15, a septal e′ velocity less than 7 cm/s, a lateral e′ velocity less than 10 cm/s, a tricuspid regurgitation (TR) velocity greater than 2.8 m/s, and an estimated systolic pulmonary artery pressure (sPAP) exceeding 35 mmHg [2].
8. LV mass and wall thickness (WT). The quantification of the myocardial mass and the measurement of the thickness allows for the identification of pathological hypertrophy. An example is hypertrophic cardiomyopathy, in which there is asymmetric LV hypertrophy with septal thicknesses above 15 mm. There are two main patterns of hypertrophy: concentric and eccentric. Concentric hypertrophy occurs in cases of chronic pressure overload such as in aortic stenosis or poorly controlled arterial hypertension. Eccentric hypertrophy is typical of volume overload—typical, for example, of aortic insufficiency or cases of dilated heart disease; the latter type of hypertrophy usually belongs to dysfunctional ventricles and therefore is a negative prognostic marker [25]. The proposed criteria for detecting structural heart disease are an LV indexed mass greater than 116/95 g/m², a relative WT exceeding 0.42, and an LV wall thickness > 12 mm [2].
9. Left atrial (LA) function. LA enlargement predicts cardiovascular risk, and an alteration of LA deformation property (strain) is a marker of negative outcomes such as cardiovascular morbidity and mortality [28]. Following the 2022 AHA/ACC/HFSA Guideline, a left atrial volume index (LAVI) equal to or exceeding 34 mL/m² is proposed as indicative of elevated filling pressures [2].
10. Advanced echocardiography. RV global longitudinal strain (RVGLS) and free-wall right-ventricular longitudinal strain (RVFWS) are two important parameters for evaluating RV function. The normal values are >−17.5% for RVGLS and >−15.3% for RVFWS. RVFWS is a more sensitive indicator of RV function since RVGLS, involving interventricular septum deformation analysis, can be influenced by LV dysfunction [29].

#### 2.1.1. Novel Approaches in TTE Evaluation for AHF

TTE is the foremost imaging modality for investigating the etiology of AHF and guiding associated therapeutic interventions [30]. Employing a “Focus Cardiac Ultrasound” (FoCUS) is recommended in the acute setting to assess LV global systolic and diastolic function, regional wall abnormalities, valvular heart conditions, and pericardial disease. Furthermore, the evaluation of right-heart structure and function, along with pulmonary pressures, carries significant prognostic implications in AHF patients [31]

Recently, lung ultrasound (LUS) has emerged as a valuable, cost-effective, portable, real-time, and radiation-free modality for detecting and monitoring pulmonary congestion in AHF patients. It surpasses the diagnostic accuracy of chest radiographs in identifying pleural and lung effusion, utilizing B-lines. The number of B-lines correlates with the severity of congestion, offering 85% sensitivity and 92% specificity for identifying cardiogenic dyspnea. The persistence of the B profile in clinically stable outpatients predicts HF-related events or mortality, providing a dynamic assessment of pulmonary congestion and response to treatment [32].

Moreover, abdominal ultrasound (AUS) has proved to be valuable in assessing inferior vena cava (IVC) diameter as an indirect measure of right atrial pressures, aiding in the early detection of abnormal intravascular volume. Additionally, AUS can identify ascites and abdominal aortic aneurysms in HF patients. Recent implementations of ultrasound techniques to assess renal blood flow offer further insights into the hemodynamic status of AHF patients [33]. Novel approaches include systemic venous ultrasonography for prognostication in AHF patients. The Venous Excess Ultrasound System (VExUS) score, incorporating IVC dilatation and the pulsed-wave Doppler morphology of hepatic, portal, intra-renal, and femoral veins, provides a comprehensive assessment of systemic congestion. Notably, an intra-renal monophasic pattern, portal pulsatility > 50%, and a VExUS score of 3 (indicating severe congestion) have demonstrated predictive value for adverse outcomes in AHF patients. The integration of these ultrasonographic findings into early and multidisciplinary follow-up visits enhances the prognostic evaluation of AHF, offering a holistic and dynamic approach to patient care [34].

#### 2.1.2. Role of Cardiac Magnetic Resonance Imaging in AHF

CMR imaging plays a fundamental role in AHF due to its high sensitivity in identifying the underlying etiology [35]. Late gadolinium enhancement (LGE) patterns help distinguish between ischemic cardiomyopathy (ICM) and non-ischemic cardiomyopathy (NICM) [36].

**Figure 1:**
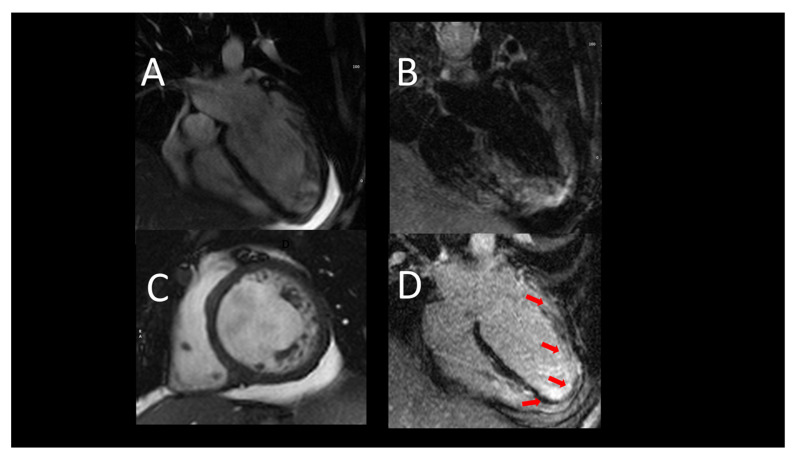
Representative case of use of cardiac magnetic resonance (CMR) in acute heart failure. (**A**) (**B**) acute heart failure, CMR images showing severe LV (left ventricle) dilatation, associated with hypertrabeculation. (**C**) T2-weight images exclude edema. (**D**) a diffuse endocardial late enhancement (red arrows) was detected, compatible with endomyocardial disease.

In ICM, LGE is transmural or subendocardial, while in a certain proportion of NICM cases, the presence of intramural or subepicardial LGE is detected. Notably, the absence of LGE does not completely exclude ICM in the case of hibernating myocardium [22]. In ICM, an important role of CMR is the assessment of myocardial viability. The presence of scars extending more than 75% of the myocardial wall indicates a low probability of recovery after revascularization. On the other hand, the presence of scars affecting less than 25% of the myocardial wall indicates a good chance of recovery [37]. In NICM, LGE has important prognostic implications in terms of site and distribution, as its extent correlates to a major number of cardiovascular events. Examples of CMR findings are shown in Figure 1.

CMR is crucial for assessing and diagnosing myocardial infiltrative diseases, such as amyloidosis, iron overload and Anderson–Fabry disease, also including rare conditions like hemochromatosis and sarcoidosis. The variability in the progression and severity of HF among individuals depends on factors such as disease subtype, organ involvement, and promptness of diagnosis and treatment [38]. While echocardiography is typically the initial imaging method for patients with HF, the utilization of CMR has grown in cases of infiltrative diseases due to its ability to reveal hypertrophy, visualize infiltration, quantify its burden, and offer potential prognostic value, with characteristic features becoming more evident in advanced stages [39]. Within cardiac amyloidosis (CA) patients, CMR holds the potential to differentiate between light chain immunoglobulin (AL) and transthyretin amyloidosis (ATTR), revealing asymmetrical LV hypertrophy (LVH) as the prevalent morphology in ATTR, often manifesting as sigmoid septum or reverse septal contour; while in AL-CA, symmetrical and concentric LVH predominates, necessitating careful differentiation from hypertrophic cardiomyopathy (HCM) or hypertensive heart disease. CMR possesses the ability to differentiate tissue properties by evaluating LGE images and quantifying cardiac amyloid burden using T1 mapping and extracellular volume (ECV) measurement [40]. CMR with LGE is also considered the preferred diagnostic method for detecting cardiac sarcoidosis involvement, revealing scar tissue and potential inflammation-related extracellular expansion. It also enables the identification of morphological abnormalities (scars and aneurisms) and the assessment of cardiac chamber function [41].

CMR stands as a pivotal diagnostic tool in guiding revascularization decisions, particularly in cases of ischemic cardiomyopathy. The degree of myocardial hyperenhancement, as detected by CMR, demonstrates a noteworthy inverse correlation with the subsequent improvement in myocardial contractility following either surgical or percutaneous revascularization procedures [42].

The great spatial resolution of CMR plays a critical role in providing precise quantification of both the extent and transmurality of myocardial scar tissue, as well as identifying viable myocardium. Notably, if the transmurality of late gadolinium enhancement (LGE) in a myocardial segment exceeds 50%, it signifies a non-viable myocardium (NVM). This serves as a crucial indicator pointing towards inadequate contractile recovery post-revascularization [43]. 

#### 2.1.3. Role of Cardiac Computed Tomography in AHF

In patients with AHF, CCT can be used to assess ventricular function when echocardiographic windows are suboptimal and CMR is contraindicated (i.e., for the presence of devices, which are particularly frequent in such patients). CCT provides a true volumetric method to assess both LV and RV size and systolic function at high spatial resolution. It can also identify typical characteristics of non-compact LV, hypertrophic cardiopathy, and arrhythmogenic RV cardiomyopathy (RV dilation and dysfunction, adipose infiltration) [44,45]. According to the 2010 Appropriate Use Criteria for Cardiac Computed Tomography, CCT angiography is considered appropriate for evaluating coronary artery disease (CAD) in patients with HF with reduced ejection fraction (HFrEF) who present a low to intermediate probability of CAD [46].

Furthermore, CCT acquisition has the potential to identify myocardial fibrosis in specific LV regions without the need for extra contrast agents or increased radiation exposure [47].

Recent validation studies have shown that CCT is capable of accurately assessing extracellular volume (ECV) in cases of cardiac amyloidosis, demonstrating good concordance with results obtained through CMR [48]. Additionally, the estimation of ECV using a single-source, single-energy CT scanner for the entire heart has proven to be both feasible and accurate. This integration of ECV measurement into a comprehensive CCT evaluation for individuals newly diagnosed with dilated cardiomyopathy can be accomplished with only a marginal rise in overall radiation exposure [49].

#### 2.1.4. Role of Nuclear Imaging in AHF

Various noninvasive imaging modalities are available for assessing biventricular function, including contrast-enhanced echocardiography, three-dimensional echocardiography (3DE), and gated heart-pool scan (GHPS) [50]. Studies exploring the concordance among these modalities in measuring LVEF and RVEF within the same patient cohort reveal discrepancies, with Pearson’s correlation coefficients ranging from 0.64 to 0.91 for LVEF and 0.27 to 0.86 for RVEF measurements [50]. This highlights the need for careful consideration in clinical management and sequential patient follow-up.

Multigated acquisition nuclear imaging (MUGA), also known as radionucleotide ventriculography (RVG) and gated equilibrium radionucleotide angiography (ERNA), emerges as a valuable third-line option when echocardiography and CMR are unavailable for LVEF assessment [51,52]. Despite its high reproducibility and minimal variability, concerns persist about radiation exposure, particularly in young patients [51,52].

Automated gated blood-pool scintigraphy (GBPS) presents itself as a potential alternative for assessing ventricular function, especially in dilated cardiomyopathy (DCM) patients. A prospective evaluation comparing GBPS with first-pass radionuclide ventriculography (FPRNV) and planar MUGA demonstrated notable correlations for LVEF values between MUGA, GBPS, and echocardiography. Strong correlations were also observed for RVEF values between GBPS and FPRNV, suggesting the routine use of automated GBPS in evaluating cardiac function in DCM patients as an alternative to traditional approaches like FPRNV [53].

Ongoing research, including modalities like MUGA, contributes to refining our understanding of their comparative utility and guides their optimal integration into cardiovascular care [50,51,52,53]. Additionally, the inclusion of modalities like MUGA enhances the cardiovascular imaging landscape, providing valuable insights for comprehensive patient care and management [54]. Stress nuclear imaging plays a crucial role in the comprehensive assessment of HF patients, providing valuable insights into cardiac function and perfusion under physiological stress. [50,51,52,53].

In HF patients, stress nuclear imaging, often performed using single-photon emission computed tomography (SPECT) or positron emission tomography (PET), helps unmask potential myocardial ischemia, assess the response of the LV under increased workload, and identify regions of impaired perfusion.

The evaluation of myocardial perfusion during stress is crucial in HF patients, where compromised blood supply can worsen existing cardiac dysfunction. Additionally, stress nuclear imaging plays a pivotal role in determining the extent of the viable myocardium, providing valuable information for informed decision-making about revascularization procedures and guiding optimal therapeutic strategies [50,51,52,53]. Furthermore, stress nuclear imaging contributes significantly to risk stratification and prognosis assessment in HF. The identification of areas with reversible ischemia and an assessment of overall cardiac performance during stress predict the likelihood of adverse cardiovascular events [50,51,52,53].

Additionally, stress nuclear myocardial perfusion imaging (SNMPI) demonstrates high sensitivity (86%) for detecting single-vessel disease, making it effective in identifying coronary artery abnormalities. It is considered the best option for patients with left bundle branch block or pacemakers causing abnormal septal motion, where other modalities might yield suboptimal results [55]

However, SNMPI involves radiation exposure, with radioactivity persisting for 3 to 4 days post-procedure. Diagnostic accuracy can be compromised by arrhythmias and soft-tissue attenuation, potentially leading to false results [55,56].

**Figure 2:**
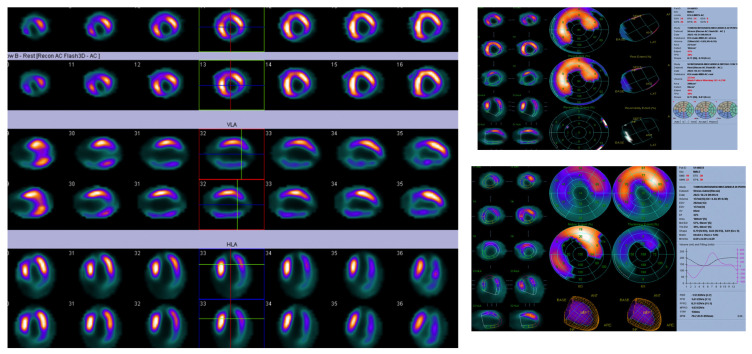
Stress/rest myocardial scintigraphy revealed the absence of myocardial ischemia but necrotic areas in the apical, inferior, and lateral walls were detected. The ejection fraction was measured at 32% after stress, and significant alterations were noted in wall motion and wall thickening in the same regions.

Stress is induced through exercise or pharmacologically using regadenoson. SPECT and PET scans offer perfusion information, with PET providing more quantitative measures and a better identification of perfusion defect location and severity. Isotope options include thallium-201 (Tl-201), technetium-99 sestamibi (Tc-99m), or technetium-99 tetrofosmin. Images are obtained at rest and after stress (Figure 2), typically taking about 20 min for each scan [55,56].

Table 1 describes the advantages, limitations, specific indications and prognostic role of different imaging modalities in AHF. 

### 2.2. Short-Term Mechanical Support

#### 2.2.1. The Intra-Aortic Balloon Pump

The intra-aortic balloon pump (IABP) consists of a percutaneously placed device that inflates in diastole, thus increasing blood flow to the coronary arteries, and deflates in systole, thus decreasing afterload. The two actions combined reduce myocardial oxygen demand and increase myocardial oxygen supply [57].

The IABP is typically placed in the cardiac catheterization laboratory under fluoroscopic guidance. However, TEE can be used to guide positioning in intubated patients in the intra-operative setting. The ideal positioning of the balloon tip is 1–2 cm distal to the left subclavian artery. This position can be confirmed by visualizing the descending aorta and then withdrawing the TEE probe until the left subclavian artery and aortic arch are visualized. Once the balloon pump is activated, the gas-filled balloon will cause shadowing and reverberation artifacts, which can be used as confirmation of the device functioning properly. In the absence of these artifacts or if bubbles are visualized in the aorta, the rupture of the IABP should be suspected. After IABP placement, although not supported by current guidelines, TTE can be used in clinical practice to monitor LV function and guide the weaning of IABP support. It can also visualize any new or worsening aortic regurgitation [58].

CCT may play a role in detecting possible complications of IABP. First, it can highlight a fearful complication—aortic dissection. Moreover, it can show the displacement of the aortic balloon or arterial embolization and organ parenchyma infarct-related soft tissue enhancement/attenuation. CMR is not indicated in monitoring possible complications. Table 2 depicts the indications and timing of echocardiographic and CCT evaluation.

#### 2.2.2. The Impella

The Impella is a rotary micro axial pump with insertion into the femoral artery and retrograde advancement up to the LV across the aortic valve: blood is aspirated from the LV and pushed into the ascending aorta. This system allows for a reduction in LV preload and an improvement in cardiac output [59]. TTE evaluation is crucial to determine if a patient is eligible for the Impella placement. The presence of severe aortic stenosis and mechanical aortic valves represent contraindications to Impella placement, whereas the presence of aortic regurgitation does not contraindicate the Impella positioning, but it should be known that regurgitation can worsen after its placement [60]. The existence of LV thrombosis represents an additional contraindication due to the potential risk of systemic embolization. Furthermore, it is essential to report the presence of conditions such as patent foramen ovale and atrial or interventricular defects, as the placement of the Impella device could potentially exacerbate a pre-existing right-to-left shunt. [61].

**Figure 3:**
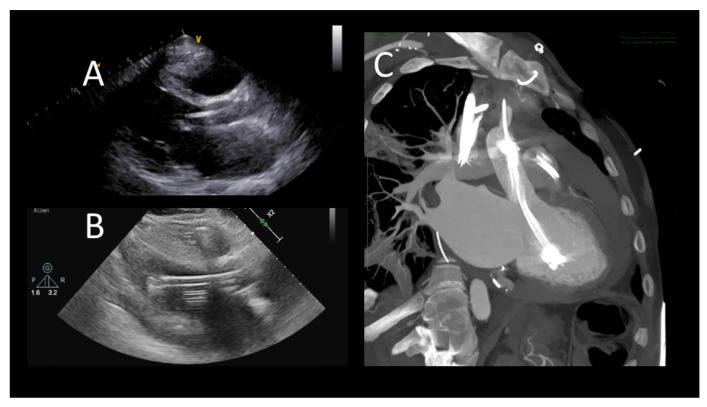
Transthoracic echocardiographic evaluation after Impella implantation (**A**) device’s correct position; (**B**) incorrect position (towards the left ventricle apex), (**C**) cardiac computed tomography showing incorrect, apical position.

As for IABP, Impella devices are commonly placed under fluoroscopic guidance, but in patients with refractory shock, preventing transportation of the patient to the cardiac catheterization laboratory, TEE can help with bedside positioning of the device [62]. One single-center study demonstrated no difference in Impella-related complications when comparing TEE-alone guided placement with the fluoroscopic guided cohort [63]. The mid-esophageal long-axis and four-chamber views can be used to visualize the guidewire crossing the aortic valve. The catheter should be oriented towards the ventricular apex. TEE can also confirm the absence of iatrogenic aortic dissection from the procedure [62]. Both TTE and TEE are helpful in identifying the correct positioning of the Impella device (Figure 3).

The distance from the aortic valve to the Impella inlet should be 3.5–5 cm, while the Impella outlet should be 1.5–2 cm above the sinuses of Valsalva [62]. Color-flow Doppler shows a mosaic pattern at the Impella inlet and outlet, further confirming its proper position. Of note, the Impella devices can migrate: in this case, the mosaic pattern will be visualized on the same side of the aortic valve [62]. Three-dimensional echocardiography can help visualize Impella positioning in comparison to other anatomical structures [64]. After placement, additional complications of the Impella placement such as damage to the mitral or aortic valve, pericardial effusion, and rupture of LV free wall must be excluded. The ideal position of the septum is median during the diastole, and displacements may indicate the presence of a right dysfunction or the need to change the speed of the Impella device. Finally, echocardiographic data can be used in conjunction with hemodynamic data to guide the weaning of the Impella by evaluating the response of the LV to progressive reduction in the support provided by the Impella (the P level). CCT plays an important role in confirming endoventricular thrombi before Impella implantation [65]. We know that the most common complications after Impella placement are hemolysis, vascular complications, bleeding, and limb ischemia. A CCT scan can identify complications such as damage to the mitral and aortic valve systems and the positioning of the device. CMR is not indicated in monitoring after Impella implantation (see Table 3).

#### 2.2.3. The Veno-Arterial Extracorporeal Membrane Oxygenation

The veno-arterial extracorporeal membrane oxygenation (V-A ECMO) system is a percutaneous system that takes over the heart and lungs. It consists of a system of inflow and outflow cannulas, a centrifugal pump, and an oxygenating membrane [66]. The ECMO provides a blood flow rate greater than 4.5 L. The effect is a noticeable reduction in LV preload increasing the afterload [67].

An echocardiographic evaluation should be performed prior to ECMO cannulation [68]. First, reversible causes of cardiovascular collapse, such as cardiac tamponade and acute valve pathology, must be excluded. The presence of an aortic dissection is a relative contraindication to ECMO positioning as it can cause an extension of the dissecting flap. The presence of aortic stenosis or mitral regurgitation should be also evaluated as they may worsen due to increased afterload due to ECMO [69]. The ECMO can be placed under fluoroscopic, TTE or TEE guidance. Usually, the venous cannula is placed in the right atrium. The mid-oesophageal bi-caval view at the TEE can easily show complications such as the passage of the cannula through the atrial septum. The arterial cannula is typically positioned within the descending aorta; TEE can confirm this location. Moreover, TEE can prevent atheromatous plaque embolization from this procedural step by referring its presence in the aorta to the operator [70].

Echocardiography also plays an essential role in assessing cardiac function when supported by the ECMO system [70]. It is important to ascertain that the aortic valve opens during systole since the high afterload due to the arterial cannula can reduce the valve opening frequency, increasing the risk of LV and aortic valve thrombosis.

Finally, echocardiography can guide ECMO weaning [71]. Echocardiographic parameters that are predictors of successful weaning are LVEF >20–25%, aortic velocity time integral (VTI) >10 cm, and lateral mitral annular systolic wave velocity (S′) >6 cm/s [71].

**Figure 4:**
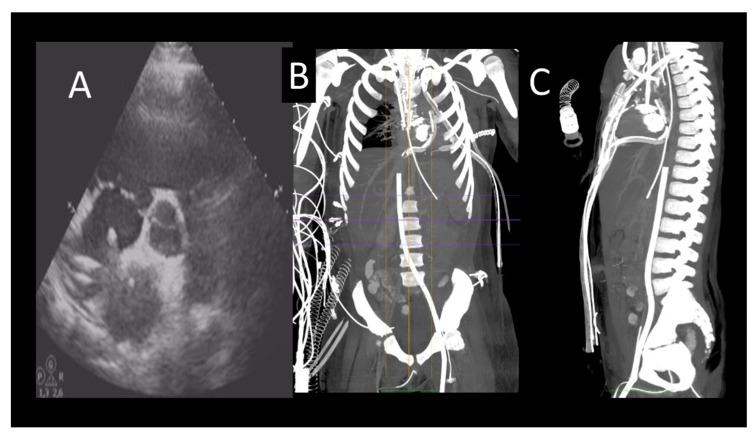
Evaluation after veno-arterial extracorporeal membrane oxygenation (VA ECMO) implantation. (**A**) Transthoracic echocardiography showing the correct position of the right atrial (RA) cannula. (**B**) Cardiac computed tomography (CCT) scan showing correct position of RA and femoral vein ECMO cannulas (**C**) Sagittal CCT showing correct position of RA and femoral vein ECMO cannulas.

The CCT scan plays a role in the identification of complications related to the placement of the ECMO. The presence of opacification defects of the arterial system is indicative of pseudo-lesion with emergent surgical indication. CCT can also be used to evaluate other complications such as cannula malposition, hematoma formation, and hemothorax (Figure 4).

An important complication of ECMO is thrombosis of the arterial system, in particular of the ascending aorta proximal to the insertion of the arterial cannula; this is mainly linked to the low flow that determines blood stasis and therefore leads to the formation of thrombi [72].

In patients with impaired RV function, there is also a predisposition to the development of pulmonary embolism [72]. Pulmonary circulation evaluation in these patients can be difficult since the contrast injected at the venous level is captured by the venous cannula before an adequate opacification of the pulmonary circulation. As a solution, the revs of the ECMO can be reduced to 500 / min for 15–25 s during contrast injection [72].

Table 4 shows the timing and role of different imaging modalities.

### 2.3. Long-Term Mechanical Circulatory Support

LVADs consist of a pump that holds the LV by receiving blood from it by means of an inflow cannula and pushing it to the level of the aorta by means of an outflow cannula. The device is placed in the mediastinum and is powered by a cable that extends abdominally to connect to a controller and a power source. There are two main types of FDA-approved LVADs: pulsatile and non-pulsatile. Those of the older generation were characterized by a pulsatile flow with a high risk of device malfunction and low survival. Heartware and Heart Mate III are characterized by a centrifugal flow; the pump is intrapericardial. Heart Mate II is characterized by an axial flow; the pump is in a pocket [73].

#### 2.3.1. Selection of LVAD Potential Candidates

TTE has a central role in the selection of the optimal candidate for LVAD implants, since it allows for evaluating (see also Table 5) [73]:

1. LVEF (particularly the demonstration of an LVEF < 25%), ventricular size and cardiac output. It may be difficult to implant patients with small LV size, especially with increased LV trabeculation.
2. The presence of intracardiac thrombi; this is not an absolute contraindication to LVAD implants, but it increases the risk of stroke during the LV cannulation procedure.
3. RV function. It is essential to evaluate the presence of signs of RV dysfunction (such as TAPSE < 18 mm, s′ < 9.5 cm/s, FAC < 35%), RV dilation, dilation of inferior vena cava, and moderate or greater tricuspid regurgitation. The presence of preoperative severe RV dysfunction may suggest the use of a biventricular MCS.
4. Valve diseases. Before LVAD implantation, it is important to detect and quantify valvular regurgitation, valvular stenosis, and prosthetic valve dysfunction. The presence of moderate to severe mitral stenosis can prevent LV cannula inflow. The presence of aortic stenosis of any severity does not affect LVAD function. In fact, LVAD bypasses the native LVOT. It is important to exclude significant aortic regurgitation (AR) before LVAD implantation because it can create a vicious cycle in which blood pumped into the aorta regurgitates into the LV. Of note, in patients with advanced HF and severe stroke volume reduction, it may be difficult to quantify aortic regurgitation. The presence of pre-operatory severe mitral regurgitation is often markedly improved after the initiation of LVAD support because of reduced LV size, reduced filling pressure and improved coaptation of MV leaflets; for these reasons, any grade of mitral regurgitation is not a contraindication to LVAD implantation. Conversely, the presence of pre-operative moderate or severe tricuspid regurgitation may indicate RV dysfunction. In patients with AV mechanical valve prostheses, reduced blood flow through the prosthesis after an LVAD implant may increase the risk of thrombosis; therefore, biological valve replacement may be considered. Finally, it is also important to exclude moderate or severe pulmonary regurgitation and pulmonary stenosis.
5. Congenital heart diseases. Some congenital common anomalies require correction before LVAD implantation. The presence of ventricular septal defects should be also excluded [73].

#### 2.3.2. LVAD Surveillance Echocardiography

**Figure 5:**
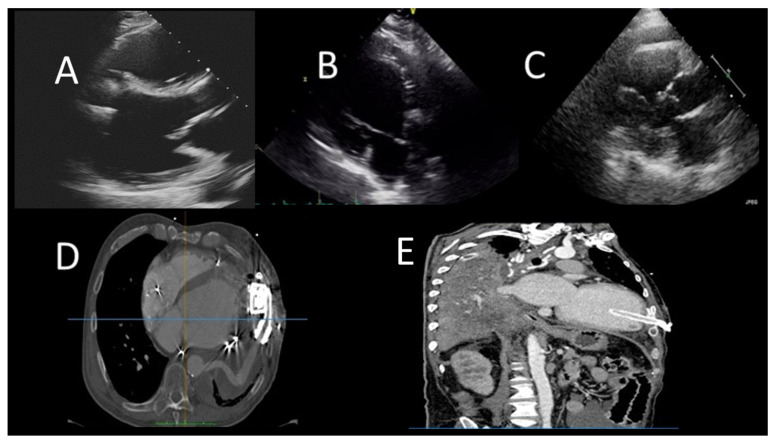
Evaluation after left ventricle assist device (LVAD) (**A**) Transthoracic echocardiography (TTE) evaluation parasternal long axis (PLAX) shows normal position of interventricular septum (IVS) and the inflow cannula; (**B**) TTEPLAX view of LVAD patient, showing incorrect right-convex position of the interventricular septum; (**C**) TTE evaluation (PLAX) of LVAD patient, showing incorrect left-convex position of the IVS; (**D**) Cardiac computed tomography (CCT) showing hematoma around LV cannula; (**E**) CCT showing small left ventricular apical thrombus.

Periodic standard TTE exams are recommended after an LVAD implant [59]. The first one is performed 2 weeks after the implant; then, they are conducted at 1, 3, 6, and 12 months post-implant and every 6 to 12 months thereafter. During the standard echocardiographic exam, it is important to evaluate and report [73]:

1. LV size and function. The most reproducible is the LV internal diameter end diastole (LVIDd) from the 2D parasternal long-axis image. The LVIDd might paradoxically be smaller than the LV internal diameter at the end of systole (LVIDs). This is a significant observation, as it is linked to excessive unloading of the LV supported by LVADs and/or severe RV dysfunction [73]. The evaluation of LVEF can demonstrate possible LV worsening or recovery. A possible complication to evaluate is LV suction with induced ventricular ectopy; this condition can be due to LV underfilling that causes the impact of inflow cannula with LV endocardium, and the solution may be speed turndown, or fluid administration in case of hypovolemia.
2. Position of interventricular septum (IVS) and cannulas. The end-diastolic IVS position may be neutral, leftward-shifted or rightward-shifted. A leftward shift can be due to elevated RV end-diastolic pressures, reduced LV preload, or LV over-decompression resulting from excessive LVAD speed. A rightward IVS shift is generally due to elevated LV end-diastolic pressures resulting from an inadequate LVAD speed setting, pump dysfunction, severe AR, or an increased LV afterload. The inflow cannula can be evaluated in the parasternal or apical TTE views. It is important to reveal the cannula’s location and orientation in relation to IVS and other LV structures. The color Doppler interrogation should demonstrate a one-directional laminar flow from LV to inflow cannula without turbulence or regurgitation. At continuous Doppler interrogation, the flow should have a peak velocity between 1 and 2 m/s; a higher velocity may suggest inflow obstruction.
3. Aortic valve (AV) opening and AR severity. It is important to evaluate the presence and the degree of AV opening because it is determined by different parameters like LVAD speed, LV native function, volume status and peripheral vascular resistance. LVAD types differ in aortic valve opening pattern, especially for the intermittent low-speed phase (e.g., 9 s for Jarvick) [74]. It is recommended that LVAD speed is set to allow at least one intermittent opening of the AV. The AV opening is assessed with M-Mode. In patients with very depressed LVEF, AV opening may not occur. When the AV remains closed, the aortic root thrombus should also be excluded. Another risk in LVAD patients is the development of AR, which is not uncommon after LVAD implantation. The assessment of its severity is partly based on careful color Doppler analysis in the parasternal long-axis view.
4. RV size and function. During TTE follow-up, RV function must be carefully evaluated. The shift of the IVS to the left side by LVADs may reduce the IVS contribution to the RV contraction. Furthermore, increased venous return created by increased cardiac output from the LVAD may worsen the RV function. This increased workload is a concern for worsening RV function that LVAD patients may already have. The classical criteria for RV dysfunction included the following parameters: TAPSE < 17 mm, tricuspid annulus systolic peak velocity (S’) velocity < 10 cm/s and RVFAC < 35% [22]. Nevertheless, the evaluation of RV function is also challenging because the correlation between RV systolic function and TAPSE and/or S’ should be considered weaker after cardiothoracic surgery.
5. Evidence of intracardiac thrombi. Recent studies on patients implanted with new-generation LVADs suggest that the LV may be a relevant site of local thrombosis and cardioembolism. Pump speed, AV opening, cannula location, and orientation are important determinants of LV flow that are drastically disrupted in LVAD patients, leading to blood stasis or abnormally large shear stresses (Figure 5) [73].

#### 2.3.3. Advanced Echocardiography in LVAD Patients

Some patients with LVADs have very difficult acoustic access in the traditional transthoracic view. Several factors influence poor image quality in LVAD patients. First, LVAD inflow and outflow cannula limit the acoustic window. Furthermore, the device may cause artifacts, and due to the device, the probe positioning during the examination may not be optimal. In such cases, ultrasound-enhancing agents (UEAs) are a good alternative—they are feasible, safe, and reproducible [75]. UEAs allow for a better definition of endocardial borders; this is useful for better quantification of the LV end-diastolic diameter and residual function. It also increases the possibility of detecting intracavitary thrombi. Moreover, it permits better visualization of the RV and helps to identify RV dysfunction and recognize patients at higher risk of RV failure. Finally, during the follow-up, UEAs can reveal the presence of pseudoaneurysms demonstrating a bidirectional flow between the pseudoaneurysm and the LV [73].

TEE is utilized to exclude right-to-left shunting and to monitor air trapping caused by the LVAD coring of the LV apex during implantation and to direct subsequent de-airing movements. TEE is also recommended in the setting of a bloodstream infection to assess vegetations and abnormal flow across the LVAD [76].

#### 2.3.4. Role of Cardiac Computed Tomography and Nuclear Imaging in LVAD Patients

CMR is contraindicated in patients with LVADs; therefore, a CCT scan represents an opportunity for a noninvasive evaluation of the function of the device and its complications [76,77,78]. A limitation of echocardiography in patients with LVADs is the incomplete visualization of the outflow cannula; the latter is well seen with the help of the CCT scan. During the follow-up, CCT can reveal complications such as compression of the right ventricle (due to pericardial clots), thrombosis, malposition, and kinking of the outflow cannula. Indications for CCT in LVAD patients include suspicion of:

(1) inflow-cannula malposition (i.e., in case of unexplained frequent LVAD suction events, recurring ventricular dysrhythmias, or residual HF due to only partial LV unloading).

(2) Pump thrombosis involving the inflow cannula or outflow tract with evidence of hemolysis.

(3) LVAD malfunction due to outflow-graft kinking, excluding an intracardiac and/or aortic root clot in patients with an unexplained transient ischemic attack or stroke.

Pre-implantation cannula placement can be optimized using CT-derived anatomy, 3D printing, and virtual modeling; nonetheless, this method needs further research [77]. Finally, whenever poor acoustic windows prevent appropriate assessment of ventricular size and function, it is possible to use either multiple-gated acquisition equilibrium radionuclide angiography or electrocardiographically gated CCT as a second-line alternative test [76].

One-fifth of LVAD recipients experience driveline infections, which can cause sepsis and/or death [78]. It has been suggested to use CT and ultrasound to detect infections in the driveline, pump, and cannula; however, due to general discoveries of metal artifacts, the usefulness of these modalities has been severely constrained. Recently, it has been shown that 18F-fluorodeoxyglucose (18F-FDG) PET/CT may be used to diagnose driveline infections in LVAD patients. Moreover, SPECT imaging has been validated to assess myocardial viability, the extension of fibrosis, and the recovery of LV function [76]. Table 6 provides the timing and role of different imaging modalities in LVAD patients.

### 2.4. Imaging in Orthotopic Cardiac Transplant (OTC)

TTE is essential in the follow-up of OTC patients. It has a role both in the immediate post-operative period and in the surveillance of short- and long-term complications [79].

Over the initial three-month period, there is an elevation in ventricular thicknesses and mass, attributed to the infiltration of inflammatory cells and graft-related edema. Prolonged persistence of ventricular hypertrophy beyond this phase could be associated with either immunosuppressive treatment or recurrent instances of acute rejection. Generally, within the first decade, LV function and regional wall motion remain intact [77]. Indeed, according to data provided by the 2019 report from the International Society for Heart and Lung Transplantation (ISHLT) registry, the occurrence of CAV after OTC was recorded at 8% after 1 year, 29% after 5 years, and 47% after 10 years [80]. An early decline in LV EF might signal either the rejection of the transplanted organ or the development of vasculopathy [79].

Diastolic function can be difficult to evaluate since cardiac denervation and the subsequent high heart rate can cause E and A wave fusion. E’ and a’ waves are of smaller amplitudes than in the normal population. A restrictive filling pattern may be present in the early post-transplant stages. Its persistence can be linked to inflammation, fibrosis and the vasculopathy of the allograft [28]. Elevated pulmonary capillary wedge pressure (PCWP) can be accurately predicted by echocardiographic signs of increased right atrial pressure (RAP) or with three out of five specific parameter values (E/A, DT, IVRT, E/E′ lateral, and Doppler PASP) exceeding cut-off values, with positive likelihood ratios between 9 and 15.3, while normal RAP or parameters below cut-off values effectively rule out elevated PCWP, supported by negative likelihood ratios ranging from 0.07 to 0.19 [81].

After cardiac surgery, the longitudinal parameters are abnormal; therefore, they are not considered sensitive parameters (including TAPSE and RV TVI). As for the atrial morphology, in the historical bi-atrial technique, an atrial enlargement and the presence of a ridge at the anastomosis are visualized. In the more recent technique, bi-caval atrial reservoir function is significantly diminished in OCT recipients, primarily associated with increased PCWP and LA enlargement, while in the RA, it is correlated with impaired longitudinal RV function [28].

Normally, valve morphology and the function of transplanted hearts are normal. There may be mild tricuspid and mitral regurgitation. Mitral regurgitation can be linked to papillary muscle edema and tends to decrease over time [63]. Tricuspid regurgitation can be detected during the first phase due to the increased pulmonary pressures, while in more advanced stages it can be linked to valve damage due to frequent biopsies or the dilatation of right chambers [79].

The presence of severe pericardial effusion leading to cardiac tamponade is rare and may be related to the presence of hearts that are smaller compared to the body surface. When a pericardial effusion is found, it is important to perform serial echocardiographic examinations (every 1–3 months) to evaluate the size, extent, and hemodynamic impact of the effusion [82].

#### 2.4.1. Advanced Echocardiography

STE may help in identifying acute cell rejection (ACR) [83]. Several studies have explored the potential of STE in detecting acute cell rejection (ACR) grade ≥2R in heart transplant recipients [84]. Promising findings include the identification of specific strain measurements, such as LV GLS and RV FWLS, as well as LV radial strain, which have shown high negative predictive values for ACR grade ≥2R [85]. Further validation in prospective trials could potentially reduce the need for frequent biopsies, especially for patients with ACR grade 2R or greater. Moreover, it has been shown that the reduction of LV torsion by at least 25% predicts, with high specificity and a high negative predictive value, ACR of at least a second degree [86].

Stress echocardiography (SE), mainly with dobutamine, is recommended in patients with a prohibitive risk for invasive coronary angiography, according to the ISHLTV guidelines. Commonly, it is acknowledged that dobutamine SE (DSE) offers initial evaluations that can help determine whether further invasive follow-up procedures are necessary [84]. A recent meta-analysis demonstrated that SE has a very low sensitivity (about 60%) in the detection of CAV and mostly cannot detect mild and moderated CAV degrees [87]). The Post-Systolic Strain Index (PSI) has been also used to evaluate CAV through DSE. To calculate PSI using specialized software, it is essential to calculate end-systolic (e-sys) and peak strain (peak-s). The PSI is calculated by finding the ratio of [peak-s–e-sys] to peak-s. If this ratio is greater than 34%, it suggests a potential presence of CAV in the patient’s heart. A study by Eroglu et al. found that this threshold has a high sensitivity of 88% in identifying patients with CAV [88].

Doppler echocardiography can be employed to gauge the velocity of blood flow within the coronary arteries and evaluate CAV, as outlined in a study by Tona et al. [89]. Coronary flow reserve (CFR) is a useful parameter, representing the maximum increase in blood flow observed between periods of rest and stress. A CFR value below 2.9, as determined by research [87], is indicative of CAV with a notably high sensitivity.

#### 2.4.2. Cardiac Magnetic Resonance

**Figure 6:**
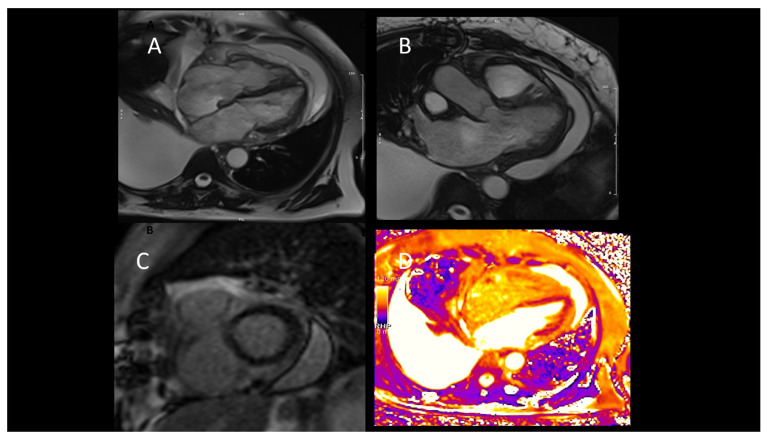
Representative case of use of CMR (cardiac magnetic resonance) in OTC (orthotopic cardiac transplantation) patients. (**A**,**B**) Immuno-mediated rejection with pericardial and pleural effusion (diastolic frame on A and B, respectively, four and three long-axis views); (**C**) Post-contrast sequences (short-axis view) demonstrated the absence of late gadolinium enhancement (LGE) and the T2-mapping was negative for inflammation (**D**). The absence of late gadolinium enhancement (LGE) and normal mapping confirmed their prognostic role since the patient demonstrated a full recovery after modification of immunosuppressive therapy.

CMR enables the early identification of rejection and CAV in patients who have undergone OCT. Emerging mapping techniques might play a role in OCT rejection diagnosis [89,90,91]. A recent study [74] showed that combining GLS > −16% and T1 time ≥ 1060 ms defined grade 1 rejection with 91% sensitivity and 92% negative predictive value, providing a potential noninvasive alternative to guide endomyocardial biopsies. Moreover, T1-mapping has demonstrated a reduction after successful treatment, serving as an excellent indicator with a negative predictive value for noninvasive rejection detection [90]. Indeed, research has demonstrated that a combined CMR strategy, incorporating both T2 mapping and extracellular volume fraction (ECV) quantification, shows a strong ability to accurately diagnose acute rejection, potentially leading to a reduction in the necessity for routine endomyocardial biopsies in these patients [91]. This multiparametric approach serves to enhance the diagnostic precision of CMR in identifying ACR [92], as shown in Figure 6. These findings were confirmed by Dolan et al. [93], who found that a combination of CMR-derived myocardial T2 and ECV holds potential as a noninvasive tissue biomarker for detecting ACR, suggesting its promise as an alternative to endomyocardial biopsy. Nevertheless, the advancement of multiparametric CMR for surveillance in transplant recipients requires additional extensive studies, particularly during instances of ACR.

Finally, stress perfusion CMR offers promise in the assessment of microvascular disease through the estimation of myocardial perfusion reserve (MPR). This CMR-based approach addresses microvasculopathy and explores its connection with myocardial perfusion reserve (MPRI) and diastolic strain rate [92]. This interesting study’s outcomes underscore the potential of CMR as a noninvasive tool for the early detection of transplant-related microvasculopathy, preceding the onset of epicardial CAV, which could potentially enhance surveillance strategies and ultimately contribute to improved patient outcomes [92].

#### 2.4.3. Cardiac Computed Tomography Angiography and Nuclear Imaging

CCT has increasingly been used to detect CAV in OTC patients [94,95,96]. Wever-Pinzon et al. in a meta-analysis of 13 studies evaluated 615 HTx patients, demonstrating a high diagnostic specificity, sensitivity, and accuracy of CCT in comparison with invasive coronary angiography (ICA) for the detection of any CAV and significant CAV using 16- and 64-slice CCT [95]. In addition, CAC > 0 was associated with an increased risk of MACE, death, and graft loss. Moreover, the absence of CAC predicted a low prevalence of International Society for Heart and Lung Transplantation (ISHLT) CAV 2–3 grade [96].

**Figure 7:**
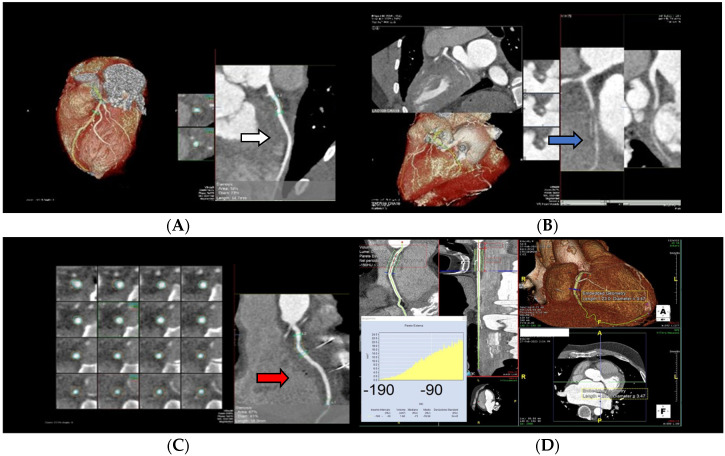
Evaluation of cardiac plaques in OTC (orthotopic cardiac transplantation) patients by CCT (cardiac computed tomography). The squares represent details of the extent of coronary stenosis. (**A**) Mild LAD (left anterior descending artery) circumferential soft plaque (white arrow); (**B**) Occlusion of LCx (left circumflex) artery (blue arrow); (**C**) Mild RCA (right coronary artery soft plaque (red arrow); (**D**) Increased pericoronary fat attenuation index values suggestive of coronary artery inflammation. Pictures from our archive.

Newer CCT technologies, such as dual-source CT and multidetector CT, increasing temporal and spatial resolution, allow for a better acquisition even at higher rate—as in the denervated transplanted heart [94,95,96]. More recently, Nous et al. [97] in a prospective observational study on 129 OTC patients demonstrated that CCT (using 2° and 3° generation dual-source CT) could be a safe and accurate alternative to ICA in CAV evaluation (Figure 7).

SPECT is a nuclear imaging method utilizing gamma-ray emissions. Several research studies have provided a range of sensitivity values (from 21% to 92%) and specificity values (from 55% to 100%) for CAV diagnosis [98]. Recent studies have generally shown improved diagnostic accuracy [99,100].

Moreover, a recent study aimed [101] to assess the effectiveness of using cadmium-zinc-telluride (CZT) SPECT with 99mTc and 201Tl tracers to measure myocardial blood flow (MBF) and myocardial flow reserve (MFR) for diagnosing CAV. The results were further compared and validated against 13 N-NH3 PET. Key findings included a strong correlation between CZT SPECT-derived stress MBF and MFR values, obtained with both 201Tl and 99mTc tracers, and those from 13 N-NH3 PET. CZT SPECT was effective in detecting low MFR (<2.0) and moderate-to-severe CAV, with results comparable to 13 N-NH3 PET.

Additionally, it is important to note that the use of PET has been investigated for CAV diagnosis, and these studies have yielded positive results [102,103]. A study by Wu et al. focused on evaluating the effectiveness of PET as a noninvasive method for detecting early stages of CAV [103]. MBF was assessed using dynamic PET, both at rest and during adenosine-induced hyperemia [103]. The researchers calculated myocardial perfusion reserve (MPR) by comparing hyperemic MBF to resting MBF. They also used a scoring system for regional PET assessments. Key findings from the study included strong correlations between MBF and MPR in different coronary artery territories. The summed stress score and summed difference score showed a moderate inverse correlation with MPR but not with intravascular ultrasound (IVUS) measurements. MPR was inversely related to plaque volume but not to maximal luminal stenosis, as determined by IVUS.

Another study [104] aimed to ascertain the simultaneous involvement of both the epicardial and intramyocardial arteries during the initial stages of CAV, highlighting that CAV is a progressive condition affecting both the epicardial and microvascular coronary systems.

One of the most clinically robust modalities used in the OTC population to screen for CAV is nuclear imaging with Rubidium-PET (Rb-PET). A recent study [105] revealed that in patients who have OTC for an extended period, there is an elevated level of resting MBF), coupled with a diminished coronary flow reserve (CFR), indicating a reduced ability to respond to stress, likely due to impaired vasodilation. This impairment is further exacerbated by the presence of CAV. Rb-PET was also shown to have prognostic significance, as serial evaluation of CFR independently predicted late mortality in OTC patients [106].

Interestingly, in a small retrospective study, quantitative coronary wall assessment and plaque analysis allowed for the early detection of CAV not detected by ICA [107]. Finally, Budde et al. demonstrated that 25% of OTC patients with focal stenosis >30% showed a low value of FFR-CT. Even without a focal stenosis, FFR-CT values were often found to be abnormal in Htx patients [108]. Table 7 describes the timing and role of different imaging modalities in OCT.

## 3. Conclusions (Take Home Messages) and Future Perspectives

This paper aims to highlight the steady advances in multimodality imaging techniques in AHF, which offer a unique opportunity for a comprehensive evaluation of such complex scenarios. In this literature review, we aim to suggest a practical, stepwise algorithm with an integrative multimodality imaging approach for the better assessment of underlying mechanisms, patterns of progression and possible complications in patients with end-stage HF and supported with short- or long-term MSD. Finally, we did not include in the present review BiVAD and TAH, aiming to provide some reflections on this future direction. Device-based therapies for HF with preserved or mildly reduced EF, encompassing atrial shunts, LV expanders, electrical and neurostimulators, and MCS devices, are still under development or used in clinical trials. These innovative approaches show promise in potentially revolutionizing HF management, offering hope for enhanced patient survival and improved quality of life. Moreover, the role of new imaging markers such as the pericoronary fat attenuation index (pFAI) in predicting cardiovascular outcomes and CAV in TCO patients should be investigated in prospective studies.

## Figures and Tables

**Table 1:** Advantages, limitations, specific indications and prognostic role of different imaging modalities in advanced heart failure (AHF).

Imaging Modality	Advantages	Limitations	Specific Indications	Prognostic Role
**Echocardiography**	Real-time, noninvasive Comprehensive assessment of heart function Various modalities (TTE, FoCUS, LUS, AUS) Dynamic assessment of pulmonary congestion	Acoustic window limitations Operator-dependent Limited in patients with poor acoustic window Limited by patient factors (obesity, COPD)	Assessment of LV/RV function, valvular conditions Detection of regional wall abnormalities Evaluation of LV mass, wall thickness Novel approaches (LUS, AUS, VExUS) for prognostication	LVGLS as predictor of mortality in HF with reduced EF Diastolic parameters for evaluating filling pressures. LA enlargement predicts cardiovascular risk
**Cardiac MRI**	High sensitivity in identifying etiology. Quantification of cardiac amyloid burden Visualize hypertrophy, infiltration, Comprehensive evaluation without contrast agent	Contraindications (claustrophobia, pacemakers) Limited availability in some settings Longer acquisition time. Limited spatial resolution	Differentiation of ICM and NICM, myocardial viability Detection of scars and inflammation in cardiac sarcoidosis Assessment of LV and RV size and systolic function Estimation of ECV for cardiac amyloidosis	Assessment of myocardial infiltrative diseases Differentiation between AL-CA and ATTR in CA patients Prognostic value in infiltrative diseases
**Cardiac CT**	True volumetric assessment Assessment of myocardial fibrosis High spatial resolution	Radiation exposure (but improving) Limited by device presence (pacemakers) Suboptimal for infiltrative diseases	Evaluation of LV and RV size and systolic function Identification of coronary artery disease in HFrEF patients Assessment of myocardial fibrosis	Identification of non-compact LV, hypertrophic cardiopathy Estimation of ECV for cardiac amyloidosis
**Nuclear Imaging**	Different modalities (MUGA, MPI with SPECT/PET) Reproducibility in LVEF assessment Potential alternatives (GBPS, SNMPI)	Radiation exposure (concerns in young patients) Limited by arrhythmias, soft-tissue attenuation Compromised diagnostic accuracy	Assessment of LVEF when echo or CMR not available Detection of myocardial ischemia, viable myocardium Assessment of cardiac function in DCM patients	Risk stratification and prognosis assessment in HF Identification of coronary artery abnormalities High sensitivity for detecting single-vessel disease

TTE = transthoracic echocardiography, LUS = lung ultrasound, AUS = abdominal ultrasound, COPD = chronic obstructive pulmonary disease, LV = left ventricle, RV = right ventricle, HF = heart failure, EF = ejection fraction, ICM = ischemic cardiomyopathy, NICM = non-ischemic cardiomyopathy, ECV = extracellular volume, VExUS = venous excess ultrasound system, CA = cardiac amyloidosis, ATTR = Transthyretin Amyloidosis, MUGA = multigated acquisition, MPI = myocardial perfusion imaging, SPECT = single-photon emission computed tomography, PET = positron emission tomography, GBPS = gated blood-pool scintigraphy, SNMPI = stress nuclear myocardial perfusion imaging, DCM = dilated cardiomyopathy.

**Table 2:** Timing and role of TTE (transthoracic echocardiography), TEE (transesophageal echocardiography) and CCT (cardiac computed tomography) in IAPB (intra-aortic balloon pump).

	TTE	TEE	CCT
Role	Monitor LV function. Guide the weaning of IABP support.	Guide positioning.	Indicated in the suspicion of complications.
Timing	Post-procedural.	Intra-procedural.	Post-procedural.
Identification of complications	New or worsening aortic regurgitations.		Aortic dissection. Displacement of aortic balloon. Arterial embolizations infarct-related soft tissue enhancement/attenuation

TTE = transthoracic echocardiography, TEE = transesophageal echocardiography, CCT = cardiac computed tomography.

**Table 3:** Timing and role of TTE (transthoracic echocardiography), TEE (transesophageal echocardiography), and CCT (cardiac computed tomography) in Impella patients.

	TTE	TEE	CCT
**Role**	Selection of candidates. Guide the placement	Selection of candidates. Guide the placement	To exclude complications.
**Timing**	Pre-procedural. Post-operative.	Pre-procedural. Intra-operative. Post-operative.	Post-procedural.
**Identification of complications**	Mitral and aortic regurgitations. Pericardial effusion. Rupture of LV free wall.	Exclude iatrogenic aortic dissection. Damage of mitralic and aortic valve.	Aortic dissection. Damage of mitral and aortic valve system.

TTE = transthoracic echocardiography, TEE = transesophageal echocardiography, CCT = cardiac computed tomography, LV = left ventricular.

**Table 4:** Timing and role of TTE (transthoracic echocardiography), TEE (transesophageal echocardiography), and CCT (cardiac computed tomography) in veno-arterial extracorporeal membrane oxygenation.

	TTE	TEE	CT
**Role**	Selection of candidates. Identification of complications. Weaning.	Guide the placement. Identification of complications.	Identification of complications.
**Timing**	Pre-procedural. Post-procedural.	Intra-procedural. Post-procedural.	Post-procedural.
**Identification of complications**	Aortic dissections. Mitral and aortic regurgitations.	Cannula malposition. Plaque embolizations. Aortic dissections. Mitral and aortic regurgitations.	Defect of opacification of arterial system. Cannula malposition. Hematoma. Hemothorax. Thrombosis of arterial system.

TTE = transthoracic echocardiography, TEE = transesophageal echocardiography, CCT = cardiac computed tomography, LV = left ventricular.

**Table 5:** Parameters to be evaluated in LVAD candidates and their influence on LVAD placement.

Parameter	Influence on LVAD Placement
EF (Ejection Fraction)	<25% indicates consideration for LVAD placement
LV Size	An adequate volume is essential to LVAD placement.
Intra-cardiac Thrombi	Exclude LVAD placement.
RV Function	Severe RV dysfunction may suggest biventricular support.
Valve Abnormalities	Significant aortic regurgitation, moderate to severe mitral stenosis, and moderate to severe tricuspid regurgitation exclude LVAD.
Congenital Heart Disease	Shunt lesions exclude LVAD placement.

LVAD = left-ventricular assist device, EF = ejection fraction, LV = left ventricle, RV = right ventricle.

**Table 6:** Timing and role of transthoracic echocardiography (TTE), advanced echocardiography, and cardiac computed tomography (CCT) and nuclear imaging in left ventricle assist device (LVAD) patients.

	TTE	TTE with Echocontrast	CT	Nuclear Imaging
Role	LV volume and function. Position of interventricular septum and cannula. RV size and function. Assessment of AV and MV	Better definition of endocardial border for quantification of LV volume and residual function. Identification of patients at higher risk of RV dysfunction.	Identification of specific complications.	Identification of specific complications.
Identification of complications	Evidence of the intracardiac thrombi.	Increases the possibility to detect intracavitary thrombi.	Compression of RV. Thrombosis. Malposition and kinking of outflow cannula.	Driveline, pump and cannula infection (PET). Assessment of myocardial viability (SPECT).

PET = Positron Emission Tomography, SPECT = Single-Photon Emission Computed Tomography.

**Table 7:** Timing and role of TTE (transthoracic echocardiography), advanced echocardiography, and CCT (cardiac computed tomography) in HT (heart transplant) patients.

	TTE	Advanced Echo	CMR	CT	Nuclear Imaging
**Role**	LV wall thickness and mass. LV volume and function. Diastolic function. Valve morphology and function. Pericardium.	LV torsion (speckle tracking). Cardiac ischemia (stress echocardiography).	LV wall thickness and mass. LV volume and function. Myocardial perfusion reserve (stress).	Coronary stenosis. Coronary plaque.	Myocardial blood flow Myocardial blood flow reserve
**Timing**	Immediate post-operative. Short-term period. Long-term period.	Short-term period. Long-term period.	Short-term period. Long-term period	Short-term period. Long-term period.	Short-term period. Long-term period.
**Identification of complications**	Allograft rejection. Primary or secondary valvopathies. Pericardial effusion.	Acute cell rejection. Cardiac allograft vasculopathy.	Acute cell rejection. Cardiac allograft vasculopathy.	Cardiac allograft vasculopathy.	Cardiac allograft vasculopathy. Microvascular vasculopathy

TTE = transthoracic echocardiography, TEE = transesophageal echocardiography, CCT = cardiac computed tomography, LV = left ventricular.

## Data Availability

Not applicable.
